# Behavioral mechanisms and learning outcomes of University Students’ GAI-assisted learning in human-AI collaboration

**DOI:** 10.1371/journal.pone.0346696

**Published:** 2026-04-28

**Authors:** Yixuan Zeng, Jing Kang, Chua Yan Piaw

**Affiliations:** Faculty of Social Sciences and Liberal Arts, UCSI University, Kuala Lumpur, Malaysia; Sultan Qaboos University and Ain Shams University, OMAN

## Abstract

With the rapid advancement of Generative Artificial Intelligence (GAI) technologies, their integration into higher education is becoming increasingly widespread. This transformation is not only reshaping students’ learning approaches but also redefining the collaborative dynamics between humans and AI. Based on the triadic framework of Exploration–Exploitation–Adaptation, this empirical study (207 valid questionnaires from Chinese university students, analyzed via structural equation modeling) investigates the behavioral mechanisms and pathways influencing learning outcomes among university students engaged in GAI-assisted learning. It examines how role adaptation, self-efficacy, task–technology fit, and institutional support affect learners’ exploration and exploitation behaviors, and how these behaviors in turn impact learning effect. The findings reveal that role adaptation and self-efficacy are the primary internal drivers of GAI-related learning behaviors, while institutional support and task–technology fit serve as essential external enablers. Both exploration and exploitation behaviors significantly enhance learning outcomes, with exploitation showing a more pronounced effect. The model demonstrates good fit and significant path relationships among variables. While the results are consistent with the proposed adaptation-exploration/exploitation-effectiveness pathway, they only reflect correlational evidence and do not establish a causal mechanism. Theoretically, this study enriches insights into human–AI collaboration in higher education. Practically, it offers guidance for the optimization of intelligent educational systems and the design of behavior-guided strategies.

## 1. Introduction

With the rapid advancement of Generative Artificial Intelligence (GAI) technologies, their application in the field of education has become increasingly widespread. GAI is gradually reshaping not only the way knowledge is delivered in university classrooms, but also how students engage cognitively with learning. [1] From text generation and image creation to coding assistance and intelligent Q&A, GAI tools have become an integral part of teaching and learning practices in higher education. Compared to traditional technologies, GAI offers superior task-handling capabilities and real-time responsiveness, and it can generate personalized content tailored to individual needs.These features provide a powerful technological foundation for the ongoing transformation toward intelligent and personalized education. [2] Against this backdrop, how learners collaborate effectively with GAI has emerged as a central issue in current educational technology research. While existing studies have explored the effectiveness of GAI in specific tasks such as knowledge generation and assignment support, [3] However, they fail to address the following critical empirical gaps: first, they have not clarified how individual psychological factors and external environmental conditions jointly drive learners’ exploratory and exploitative behaviors in Generative Artificial Intelligence (GAI)-assisted learning; second, the differential mechanisms through which these two types of behaviors independently or interactively impact learning outcomes remain unexamined; third, existing behavioral frameworks have overlooked the adaptive process that mediates the relationship between individual-environment interaction and behavioral choices. In reality, whether engagement with GAI is associated with improved perceived learning performance largely depends on whether learners can move from mere initial exposure to meaningful, in-depth use at cognitive, emotional, and behavioral levels [4,5].

To address the aforementioned research gap, this study adopts the Exploration–Exploitation–Adaptation triadic behavioral framework to examine how learners in higher education respond to GAI-assisted learning and how such responses influence their learning performance. Specifically, the study aims to explore the following key questions:

(1) How do role adaptation, self-efficacy, task-technology fit, and institutional support influence learners’ exploration and exploitation behaviors in Generative Artificial Intelligence (GAI)-assisted learning?
(2) What are the differential effects of exploration and exploitation behaviors on learning effect?
(3) To what extent do exploration and exploitation behaviors play a mediating role between the four antecedent factors and learning effect?

Compared to alternative frameworks, this one offers unique advantages: for instance, the Technology Acceptance Model (TAM) focuses only on “acceptance intention” and fails to address dynamic behavioral transitions in GAI usage, while the Information Foraging Theory (IFT) overlooks the adaptive process linking individual-environment fit to behaviors. In contrast, our framework systematically integrates behavioral patterns and adaptive mechanisms, making it more suitable for unpacking complex human-AI synergy mechanisms in learning scenarios.

Drawing on four dimensions—role adaptation, self-efficacy, task–technology fit, and institutional support—this study constructs a structural equation model to empirically test the influence of each variable on exploration and exploitation behaviors, as well as their subsequent impact on learning performance. The findings aim to uncover the core mechanisms through which GAI empowers learning, offering both theoretical insight and practical guidance for optimizing intelligent educational systems and developing behavior-oriented strategies in higher education.

## 2. Theoretical framework and literature review

### 2.1. Theoretical evolution of human–AI collaborative education

Against the backdrop of rapid technological advancement, the iterative development of artificial intelligence is driving profound transformations in education. The core logic of human–AI collaborative education has evolved from “instrumental substitution” to “cognitive complementarity”: [6] humans contribute ethical judgment and contextual understanding, while machines undertake knowledge computation and pattern recognition, together forming a synergistic relationship oriented toward co-creation.

At present, human–AI collaborative education has entered a “collaborative creation stage” dominated by generative AI (since 2022), in which GAI has shifted from a merely supportive tool to a “cognitive collaborator”. [7] This transformation is exemplified in the “Learner–GenAI–Collective Knowledge” triadic model proposed by Yong-Gui and Yu-Ting, [8] where GAI, through mechanisms of knowledge sharing, argumentative negotiation, and co-evolution, becomes a key hub linking individual cognition with collective intelligence. Empirical evidence further indicates that this collaborative model significantly enhances students’ collaborative problem-solving (CPS) abilities and team creative performance (TCP) in complex tasks such as digital creation. [7,9]

In recent years, theoretical breakthroughs in human–AI collaborative education have primarily focused on the construction of triadic subject structures and the reconfiguration of cognition. At the relational level, the traditional teacher–student dyad has evolved into a triadic structure of teacher–intelligent agent–student, in which the intelligent agent attains a quasi-subject status through its capabilities for reasoning and affective interaction. [10,11] Under this new structure, intelligent agents are differentiated into three functional roles: lesson preparation, instructional delivery, and post-lesson reflection.From an epistemological perspective, there has been a marked shift from static knowledge systems to dynamically generated knowledge. For instance, large AI models can now generate contextualized cases in real time and construct interdisciplinary knowledge graphs, thereby facilitating the transformation of educational resources from pre-defined content to open and adaptive knowledge ecologies. [12] This “quasi-subject” status has direct empirical significance for the subsequent research model. It enables learners to redefine their roles—from passive recipients of knowledge to active constructors who collaborate with AI—while such role reconstruction constitutes a critical prerequisite for shaping learners’ exploration and exploitation behaviors. At the level of learning mechanisms, GAI functions as an external knowledge carrier that deepens distributed cognition through three key capacities: “memory outsourcing” (knowledge storage), “cognitive visualization” (the display of problem-solving chains), and “metacognitive scaffolding” (learning monitoring). [13] These functions not only reduce the cognitive load associated with exploring new knowledge, but also provide structured support for the precise and efficient use of tools in task completion, thereby directly mapping onto the core constructs of exploration behavior and exploitation behavior.

### 2.2. The pedagogical adaptation of the exploration–exploitation framework

The Information Foraging Theory (IFT) and the Exploration–Exploitation framework, both originating from computer science and behavioral ecology, represent a significant breakthrough in the interdisciplinary integration of theories when applied to higher education contexts. Proposed by Pirolli and Card in 1995, IFT draws on the optimal foraging theory in biology to conceptualize how individuals maximize knowledge acquisition efficiency by balancing perceived value and cognitive cost within information environments. At its core, IFT analogizes learners’ information-seeking behaviors to a predator–prey dynamic, where individuals must constantly strike a balance between exploring unknown knowledge domains (exploration) and delving deeper into familiar domains (exploitation) in order to optimize cognitive gains. Once introduced into the field of educational technology, this framework rapidly gained prominence as a key lens through which to examine the collaborative mechanisms between humans and Generative Artificial Intelligence (GAI). It provides a theoretical foundation for understanding how learners navigate GAI-supported environments by dynamically shifting between exploratory and exploitative strategies.

The Exploration–Exploitation framework, originally derived from organizational learning theory, Proposed officially by American organizational theorist James G. March in 1991, this theory is grounded in the perspective of organizational learning and clarifies two core behaviors for organizations to adapt to their environments: Exploration refers to pursuing new possibilities, encompassing breakthrough activities such as searching and innovation; Exploitation involves optimizing existing resources, manifested as incremental behaviors like efficiency improvement and process refinement. [14] It is adapted in this study as an analytical tool for educational contexts. In the context of Generative AI (GAI), exploration behavior (a learning strategy for knowledge expansion) is manifested through open-ended prompts or socially constructed keywords to expand knowledge boundaries；exploitation behavior (a learning strategy for knowledge deepening) is manifested through iterative refinement of precise prompts to conduct in-depth inquiry within a specific knowledge domain, emphasizing problem-solving precision.Kallunki et al. [15] found that when educators engage with GAI, exploratory and exploitative prompts are generally balanced. Their study also revealed a significant positive correlation between exploration behavior and topic diversity. However, over-reliance on GAI-generated keywords can lead to topic narrowing effects, highlighting the inherent tension between exploration and exploitation in human–AI collaboration. From the learners’ perspective, Wing’s [16] concept of computational thinking—emphasizing abstraction, decomposition, and algorithm design—offers a methodological foundation for effectively navigating GAI. In collaborative scenarios, learners must employ recursive thinking to analyze complex problems, use abstraction to convert open-ended questions into manageable keywords, and rely on algorithmic processes to co-construct solutions with AI.For example, law students can refine keyword specificity to guide GAI in generating diverse analytical frameworks, and then apply their critical thinking skills to evaluate and select the most appropriate responses. This process exemplifies the computational thinking pattern of decomposition–abstraction–algorithmization, [17] enabling productive synergy between human cognition and artificial intelligence. In this study, exploration and exploitation are essentially learners’ learning strategies—exploration focuses on knowledge expansion and cognitive innovation, while exploitation emphasizes knowledge deepening and task optimization. Prompt design, functional operations, and similar actions are considered tool-use behaviors, which serve as the concrete means for implementing these strategies, rather than the strategies themselves.

Exploration and exploitation behaviors must be examined within the triadic interaction system of teacher–student–GAI. Recent studies suggest that GAI not only transforms the way information is accessed but also reshapes the fundamental nature of pedagogical relationships. The teacher’s role is shifting from that of a knowledge transmitter to a designer of prompts and a facilitator of cognitive dissonance. By intentionally creating divergences between human and AI-generated perspectives, teachers can stimulate learners’ critical reflection and deeper engagement with content. [18]

### 2.3. Multilevel integration of adaptation dimensions

From the perspective of human–AI collaboration, adaptation in higher education with GAI specifically refers to the dynamic process by which learners, teachers, and institutions actively adjust cognition, behavior, and organizational policies to accommodate GAI-enabled collaborative scenarios. This construct is clearly distinguished from self-efficacy (individuals’ subjective beliefs about their own capabilities) and task–technology fit (TTF, the objective alignment between technological functions and task requirements). adaptation emphasizes active adjustment, whereas the latter focus on beliefs in ability and objective fit, respectively.

Research shows that teachers optimize course design and assessment practices to balance efficiency and trust. Sutedjo et al. [19] found that embedding fact-checking and explanatory interaction mechanisms can enhance teachers’ trust and control over GAI. Lyu et al. [20] further note that even teachers who are proficient in using GAI employ trust-calibration strategies to adjust their teaching standards. Students enhance critical thinking and writing skills through iterative “generate–review–regenerate” cycles while maintaining cautious scrutiny of GAI accuracy; for example, engineering students acknowledge that GAI improves learning efficiency and creativity but remain cautious about its impact on actual academic performance. [21] At the group level, GAI reshapes collaboration patterns and role division. AI assistants such as Harvard’s CS50 Duck facilitate iterative “generate–co-create–feedback” cycles, with student teams employing reflective logs to mitigate overreliance risks. [22] At the organizational level, universities have implemented GAI ethical guidelines and specialized training programs, integrating AI literacy into course design, assessment standards, and academic integrity systems, thereby achieving institutional adaptation [23].

**Fig 1:**
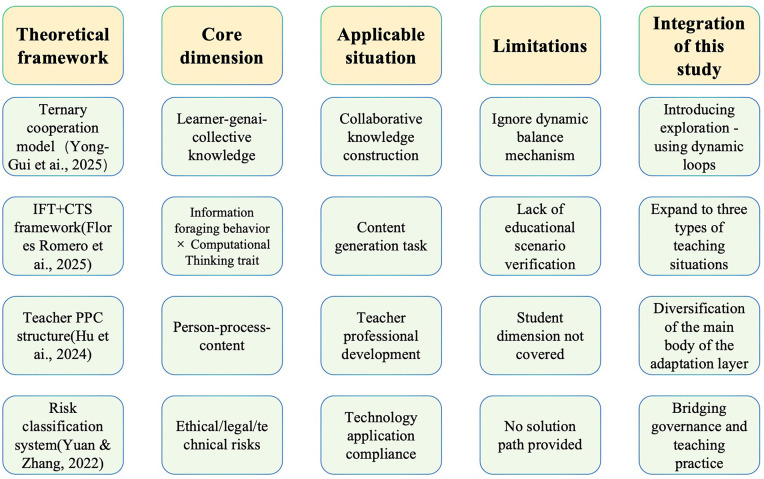
Key Theoretical Frameworks in Human–AI Collaborative Education Research.

Despite these advancements, existing studies often overlook the critical dimension of adaptation in human–AI collaboration, resulting in a misalignment between technological implementation and pedagogical needs. To address this gap, the present study proposes a triadic framework of Exploration–Exploitation–Adaptation, grounded in a comparative analysis of key theoretical models in human–AI collaborative education (see Fig 1).This study integrates perspectives from both Task–Technology Fit (TTF) and Role Adaptation, emphasizing the dynamic alignment between technological functions and user roles. For instance, Hu et al. [24] introduced the “Teacher PPC Structure”, which analyzes teacher professional development from three dimensions—Person, Process, and Content—and provides a foundation for individual-level adaptation. Meanwhile, Yuan and Zhang [25] proposed a “Risk Classification System” that addresses ethical, legal, and technological risks, offering theoretical support for institutional-level adaptation.

## 3. Research framework and hypotheses

### 3.1. Theoretical foundations and model construction

Guided by the key theoretical frameworks of human–AI collaborative education, this study proposes a triadic “Exploration–Exploitation–Adaptation” model. Drawing upon Task–Technology Fit (TTF) Theory, Self-Efficacy Theory, and Institutional Support Theory, an integrated research framework is constructed to examine the underlying collaborative mechanisms.

The model is structured into three hierarchical levels:

Fit Dimension: This level includes four variables—task–technology fit, role adaptation, self-efficacy, and institutional support—which together reflect the degree of alignment between GAI technologies and users’ roles and environments.

Dualistic Behavior: This layer comprises two forms of user engagement with GAI—exploration behavior, referring to open-ended experimentation with new technologies; and exploitation behavior, referring to the stable, task-oriented use of these technologies.

Result Output: The final layer represents the learning performance outcomes, including knowledge acquisition, skills enhancement, and cognitive development.

In this study, interaction or moderation effects are not included because the focus is on revealing the core mechanisms of GAI-assisted learning; including complex effects could obscure the primary causal pathways. These effects may be explored in future research. Similarly, teacher-level variables are not incorporated, as the study emphasizes learner-driven human–AI collaboration. The influence of teachers is indirectly captured through existing constructs, such as guidance within institutional support and instructional task design within task–technology fit, thereby maintaining focus on the core issue of learner behavioral adaptation.

### 3.2. The relationship between adaptation dimensions and dual behaviors

#### 3.2.1. The impact of task–technology fit on exploration and exploitation behaviors.

Task–Technology Fit (TTF) Theory [26] posits that when the technological tools used by individuals are well-aligned with the tasks they are performing, both their intention to use and behavioral efficiency are significantly enhanced. Saifi et al. [27] further emphasized that TTF is strongly influenced by the characteristics of the task and the features of the technology involved. In the context of this study, TTF is interpreted within GAI-supported teaching and learning environments. When teachers or students perceive that AI tools effectively support educational goals—such as content generation, instructional design, or problem-solving—they are more likely to engage in active exploration behaviors and consistent exploitation behaviors.Accordingly, the following hypotheses are proposed:

H1: Task–technology fit has a significant positive effect on exploration behavior.

H2: Task–technology fit has a significant positive effect on exploitation behavior.

#### 3.2.2. The impact of role adaptation on exploration and exploitation behaviors.

Role adaptation refers to the process by which individuals redefine their roles and responsibilities in response to new technological contexts. According to the extended Technology Acceptance Model, [28] this concept can be applied to understand how teachers and students adjust to the integration of GAI in educational settings. When users are able to clearly identify their evolving roles—such as AI-assisted instructional designers or AI feedback interpreters—they are more likely to engage in proactive exploration and effective exploitation of AI tools.Accordingly, the following hypotheses are proposed:

H3: Role adaptation has a significant positive effect on exploration behavior.

H4: Role adaptation has a significant positive effect on exploitation behavior.

#### 3.2.3. The impact of self-efficacy on exploration and exploitation behaviors.

Self-efficacy is widely regarded as a key psychological construct influencing individuals’ behavioral choices and persistence when encountering new technologies. [29] In a seminal study, Compeau and Higgins [30] found that computer self-efficacy significantly affects users’ expectations of outcomes, emotional responses (such as enjoyment and anxiety), and actual usage behaviors.In the context of this study, self-efficacy refers to the confidence of teachers and students in their ability to effectively operate GAI tools. Higher levels of self-efficacy are expected to encourage more in-depth technological exploration and sustained usage. In teaching and learning environments, self-efficacy not only influences the frequency of technology trials but also the depth of integration into pedagogical or academic practices.Accordingly, the following hypotheses are proposed:

H5: Self-efficacy has a significant positive effect on exploration behavior.

H6: Self-efficacy has a significant positive effect on exploitation behavior.

#### 3.2.4. The impact of institutional support on exploration and exploitation behaviors.

Organizational policies and environmental support—such as technical training, policy incentives, and resource platforms provided by higher education institutions—are key external factors that promote the adoption of new technologies by both teachers and students. [31] Institutional support can lower the threshold for technology useand enhance users’ willingness to engage, thereby encouraging both exploration and exploitation behaviors. [32] Accordingly, the following hypotheses are proposed:

H7: Institutional support has a significant positive effect on exploration behavior.

H8: Institutional support has a significant positive effect on exploitation behavior.

### 3.3. The relationship between dual behaviors and learning performance

In this study, dual behaviors emphasize two distinct but complementary learning strategies. Exploration behavior—such as trying new strategies and engaging in deeper interactions—can lead to cognitive transfer and innovation. [14] In contrast, exploitation behavior—such as efficiently completing instructional tasks—helps to consolidate knowledge structures and skill mastery. In GAI-supported learning environments, exploration behavior may stimulate students’ critical thinking and creativity, while exploitation behavior enhances learning efficiency and the quality of academic outcomes. Exploration behavior refers to learners’ engagement in trying new tools, experimenting with strategies, collaborating with peers, and identifying novel problems. For example, Chang et al. [33] found that GAI-based exploratory writing training significantly improved students’ metacognitive skills and creative thinking abilities.Accordingly, the following hypothesis is proposed:

H9: Exploration behavior has a significant positive effect on learning performance.

Exploitation behavior, by contrast, involves efficient task completion and optimal use of resources under established norms. In GAI-assisted contexts, this refers to students’ effective integration of AI tools into their academic work—for example, using GPT for grammar refinement or idea polishing. Such behavior enhances both learning efficiency and output quality, allowing students to achieve better knowledge acquisition and performance outcomes within limited timeframes. Li et al. [34] demonstrated that exploitation-oriented task engagement with GAI significantly improved students’ writing quality and academic achievement in complex writing tasks.Accordingly, the following hypothesis is proposed:

H10: Exploitation behavior has a significant positive effect on learning performance.

### 3.4. Research model diagram

**Fig 2:**
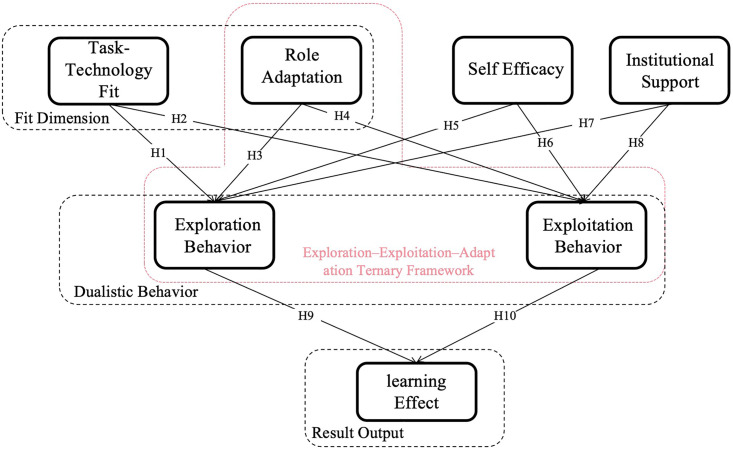
Proposed Research Model.

Based on the theoretical foundations and the proposed variable relationships, the research model is constructed as shown in Fig 2. The model comprises four antecedent adaptation variables, two mediating behavioral variables, and one outcome variable, reflecting a causal chain from adaptation to behavior to learning outcomes. This structure facilitates a deeper examination of the collaborative mechanisms and impact pathways through which GAI technologies are integrated into teaching and learning processes in higher education.

## 4. Data collection and analysis

### 4.1. Scale design

Based on the Exploration–Exploitation–Adaptation triadic framework, this study developed a questionnaire to systematically examine the collaborative mechanisms and effectiveness of Generative Artificial Intelligence (GAI) in higher education learning contexts. The scale consists of eight core dimensions, namely: demographic information, task–technology fit, role adaptation, self-efficacy, institutional support, exploration behavior, exploitation behavior, and learning performance, with a total of 39 items (see Table 1). All constructs in the questionnaire were adapted from established scales in the literature. [35–40] Some items were appropriately revised to better align with the real-world instructional environment of Chinese universities and the mainstream GAI tools used (e.g., ChatGPT, Wenxin Yiyan), in order to enhance the contextual relevance and measurement validity of the instrument.

**Table 1:** Core Dimensions of the Questionnaire Scale.

Model Dimension	Number of Items	Code Range	Literature Sources
Basic Information	4	Q1—Q4	Self-compiled
Task Technology Fit(TTF)	5	Q5—Q9	[35]
Role Adaptation(RA)	5	Q10—Q14	[36]
Self Efficacy(SE)	5	Q15—Q19	
Institutional Support(IS)	5	Q20—Q24	[37]
Exploration Behavior(EB1)	5	Q25—Q29	[38–40]
Exploitation Behavior(EB2)	5	Q30—Q34	
Learning Effect(LE)	5	Q35—Q39	

The survey items were adapted strictly in line with the core constructs of the original scales. Wording was slightly adjusted to fit the context of Chinese higher education and commonly used GAI tools, ensuring that construct validity was preserved. The scales were translated and back-translated by bilingual experts, with semantic consistency checks performed to guarantee linguistic equivalence. Exploration and exploitation items were defined around distinct core dimensions—“knowledge expansion and functional novelty” for exploration, and “task deepening and process optimization” for exploitation—thereby avoiding overlap between the two constructs.

Among them, task–technology fit, role adaptation, self-efficacy, and institutional support are treated as predictor variables, assessing whether learners possess the technical readiness and psychological preparedness to effectively integrate GAI tools into their learning tasks. Exploration behavior and exploitation behavior serve as the dual behavioral variables, measuring the extent to which learners tend to either experiment with new GAI functions or optimize existing processes. Learning performance functions as the outcome variable, reflecting the overall impact of GAI on learning efficiency, depth of understanding, and quality of task completion.

All items were rated using a 7-point Likert scale (1 = “Strongly Disagree”, 7 = “Strongly Agree”), which facilitates the quantitative analysis of the data and the application of Structural Equation Modeling (SEM) in subsequent analysis (see Table 2).

**Table 2:** Measurement Items and Sources.

Model Building	code	Questionnaire Questions	Literature Sources
Basic Information	Q1	Identity:□ Undergraduate student □ Master’s student □ Doctoral student □ Teacher □ Academic administrator □ Other: ______	
	Q2	Duration of GAI usage:□ < 3 months □ 3–6 months □ 6–12 months □ 1–2 years □ > 2 years	
	Q3	Commonly used GAI tools (select all that apply):□ ChatGPT □ Wenxin Yiyan (Ernie Bot) □ Deepseek □ Doubao □ Other: ______	
	Q4	Academic field:□ Humanities and Social Sciences □ Science and Engineering □ Business □ Medicine □ Art and Design □ Education □ Other: ______	
Task-Technology Fit	TTF1(Q5)	GAI has provided strong support for my learning tasks.	[35]
	TTF2(Q6)	Using GAI effectively meets the needs of my academic tasks.	
	TTF3(Q7)	I believe my learning tasks are well matched with the capabilities of GAI tools.	
	TTF4(Q8)	I can smoothly integrate GAI into my daily learning activities.	
	TTF5(Q9)	I can understand and operate most of the functions provided by GAI.	
Role Adaptation	RA1(Q10)	When I encounter difficulties using GAI, I can try different ways to solve them.	[36]
	RA2(Q11)	I am able to adapt my role to fit into GAI-supported learning scenarios.	
	RA3(Q12)	GAI has helped me rethink my role in the learning process.	
	RA4(Q13)	Even when facing challenges, I am capable of continuing to use GAI to complete tasks.	
	RA5(Q14)	I can flexibly adjust my usage strategies based on how GAI performs.	
Self Efficacy	SE1(Q15)	I am confident in my ability to use GAI tools efficiently.	
	SE2(Q16)	Even without guidance from others, I can independently use GAI to complete learning tasks.	
	SE3(Q17)	I believe I can handle different types of GAI tools.	
	SE4(Q18)	I am able to identify and manage the learning risks associated with GAI.	
	SE5(Q19)	I am capable of evaluating the relevance and validity of GAI-generated outputs.	
Institutional Support	IS1(Q20)	My university provides specialized training to help me use generative AI tools proficiently.	[37]
	IS2(Q21)	I can easily access online or offline tutorials and guidance on how to use GAI.	
	IS3(Q22)	Teachers or advisors actively help me resolve issues encountered when using GAI.	
	IS4(Q23)	My university provides technical resources for GAI, such as access permissions and system integration.	
	IS5(Q24)	I can receive timely feedback and support when using GAI.	
Exploration Behavior	EB1(Q25)	I often explore different functions of GAI tools.	[38–40]
	EB2(Q26)	I am willing to use GAI proactively for learning, even without being required to do so.	
	EB3(Q27)	I enjoy using GAI tools to try new methods or complete new types of tasks.	
	EB4(Q28)	I have learned knowledge or skills that I previously did not understand through GAI.	
	EB5(Q29)	I experiment with different prompts to explore how GAI responds.	
Exploitation Behavior	EB1(Q30)	I often use GAI tools when completing academic assignments.	
	EB2(Q31)	I repeatedly use GAI to improve the expression in my academic writing.	
	EB3(Q32)	I have incorporated GAI into my daily learning routines.	
	EB4(Q33)	GAI plays an important role in completing my learning tasks.	
	EB5(Q34)	I use GAI to enhance my ability to solve complex problems.	
Learning Effect	LE1(Q35)	Using GAI has improved my learning efficiency in courses.	
	LE2(Q36)	GAI has helped me gain a deeper understanding of complex concepts.	
	LE3(Q37)	The quality of my assignments or reports has improved after using GAI.	
	LE4(Q38)	With the support of GAI, I can complete learning tasks more quickly.	
	LE5(Q39)	Overall, GAI has had a positive impact on my learning outcomes.	

### 4.2. Data collection

This study employed a questionnaire survey as the primary method of data collection. The questionnaire was developed based on the Exploration–Exploitation–Adaptation triadic framework, and comprised eight modules. In addition, the demographic section included background variables such as respondent identity, duration of GAI usage, commonly used GAI tools, and academic discipline, which were designed to support subsequent heterogeneity analyses.

Participants were recruited between July and August 2025. The study involved an anonymous online questionnaire and did not collect any personally identifiable or sensitive information. Participation was entirely voluntary. In accordance with the ethical policy of UCSI University and international standards (e.g., British Educational Research Association, 2018), this study was reviewed and determined to be exempt from full institutional ethical review by the Faculty of Social Sciences and Liberal Arts, UCSI University, Kuala Lumpur, Malaysia, on 8 June 2025. As this study was granted an exemption, no formal approval number was issued in accordance with institutional policy. Informed consent was obtained electronically from all participants prior to the commencement of the questionnaire.

The survey was distributed online via the SoJump platform and disseminated through university student forums, faculty WeChat groups, and academic collaboration networks. A total of 215 responses were received. Invalid questionnaires were excluded if the completion time was less than 180 seconds, if logical contradictions were present (e.g., simultaneously selecting “undergraduate” and “doctoral student” as academic status), or if the respondent reported no actual experience using GAI tools, 207 valid questionnaires were retained, yielding a valid response rate of 96.28%.In terms of respondent identity, doctoral students accounted for the largest proportion (58.94%), followed by master’s students (25.6%) and undergraduates (11.11%). A small number of respondents were teachers (3.86%) or teaching administrators (0.48%). This sample structure indicates a predominance of senior students, who typically demonstrate stronger learning autonomy and a more established foundation in using GAI tools. This composition is conducive to an in-depth examination of changes in learning behaviors and adaptation strategies. However, it should be noted that the concentration of higher-level learners may limit the direct generalizability of the findings to the broader undergraduate population. Future studies should expand the proportion of undergraduate participants to enhance the universality of the results. Regarding GAI usage experience, over 70% of participants had used GAI tools for more than one year, and 50.24%had more than two years of usage experience. These figures suggest a user group with relatively deep engagement and accumulated familiarity, appropriate for investigating the practical value of GAI in academic tasks.

Given that this study employed Structural Equation Modeling (SEM) as the core analytical method, the sample size was determined in accordance with the established requirements for SEM-based model estimation. According to methodological guidelines, the required sample size in SEM should be proportional to the model’s complexity, with a widely accepted rule suggesting that the sample should be 5–10 times the number of observed variables, in order to ensure the stability of parameter estimates and the reliability of model fit. [41] Moreover, previous research has indicated that while larger samples can increase statistical power, sample sizes exceeding 500 may lead to oversensitivity in chi-square statistics, potentially resulting in misleading fit indices and biased significance judgments. [42] Accordingly, the 207 valid responses collected and analyzed in this study not only meet the minimum requirements for SEM, but also help avoid distortion caused by an excessively large sample size. This sample size ensures both statistical adequacy and representativeness, making it appropriate for robust SEM analysis.

In terms of commonly used GAI tools, ChatGPT was identified as the most widely adopted platform (66.18%), followed by Deepseek (53.14%), Doubao (36.23%), and Wenxin Yiyan (28.5%). This suggests that respondents demonstrated a considerable degree of diversity in platform usage, though there was limited adoption of “other” tools, indicating a clear concentration around leading GAI platforms in the current market.Regarding disciplinary background, the majority of respondents came from art and design fields (26.57%), education-related disciplines (25.6%), and the humanities and social sciences (17.87%). Additional representation was observed from science and engineering, medicine, and business majors, reflecting the cross-disciplinary applicability and penetration of GAI technologies. Notably, in disciplines such as art, education, and the humanities, GAI’s capabilities in language generation, knowledge reorganization, and task support offer substantial collaborative value.Overall, the collected dataset demonstrates a high degree of diversity and representativeness across user identities, levels of GAI experience, platform usage, and academic fields. This provides a solid empirical foundation for the subsequent analysis and enhances the practical relevance of the study’s findings.

### 4.3. Data analysis procedure

The data analysis in this study followed a systematic five-step procedure to ensure scientific rigor and reliability of the results. First, after collecting survey responses, data were cleaned based on explicit criteria, including completion time less than 180 seconds, logical inconsistencies, or lack of actual GAI experience. Second, the reliability and validity of the scales were assessed using composite reliability (CR), average variance extracted (AVE), and the Fornell-Larcker criterion to ensure measurement stability and discriminant validity. Third, a structural equation model was constructed in AMOS 26.0, with overall model fit evaluated using key indices such as CMIN/DF, CFI, and RMSEA. Fourth, the significance of path coefficients between variables was tested to verify the hypothesized causal relationships. Fifth, the R² values of endogenous latent variables were calculated to assess the model’s explanatory power for variable variance. This stepwise procedure provides a solid methodological foundation for testing the study hypotheses.

### 4.4. Reliability and validity analysis

All data in this study were collected through a structured self-report questionnaire, relying primarily on learners’ self-perceptions and subjective responses. This single-source, single-method approach may give rise to common method variance (CMV), potentially introducing systematic bias into the observed relationships among variables. [43] To mitigate the risk of CMV, several procedural remedies were implemented at the questionnaire design stage. Specifically, following the recommendations of Shiau et al. [44], the measurement items for different latent constructs were distributed across separate pages, allowing participants brief intervals between item groups and thereby disrupting cognitive continuity. This design was intended to reduce habitual response patterns and minimize serial bias. In addition, item sequences within the questionnaire were randomized, further controlling for response style effects and enhancing data quality. It should be noted that this study did not use other statistical methods (such as Harman’s single-factor test or control variable approaches) to assess CMV. However, the procedural design described above has largely controlled for common method bias, thereby enhancing the data’s validity and the interpretability of the model results.

The aforementioned procedural controls are fully sufficient to address CMV in this study. First, the item separation and randomization strategies are well-established effective methods to alleviate common method bias in self-reported questionnaire research. Second, all latent variables in this study present excellent reliability (CR > 0.90) and discriminant validity (AVE square root > inter-construct correlations), which fundamentally restrict the overestimation of variable relationships caused by CMV. Third, this study adopts structural equation modeling with strict model fit evaluation, and the stable path coefficients and fit indices indicate that CMV does not interfere with the core research conclusions. Therefore, additional statistical tests for CMV are not required.

The reliability of the questionnaire was assessed using Structural Equation Modeling (SEM) (see Table 3). The results showed that the composite reliability (CR) for all latent variables exceeded 0.90, well above the commonly accepted threshold of 0.70, [45] indicating strong internal consistency among items within each dimension. The CR values in this study are relatively high, primarily because the survey items were carefully adapted to align with the core meaning of each dimension and optimized for the context of GAI use in Chinese higher education, reducing misunderstanding and increasing inter-item correlations. Each dimension contains only five items, with clear boundaries and no cross-dimensional semantic overlap. Regarding potential item redundancy, comparisons of related items (e.g., RA1 vs. RA3, SE1 vs. SE3) show that while they are semantically related, they capture different aspects within their respective dimensions. The high AVE values further indicate that convergent validity was not compromised. At the item level, all standardized factor loadings were above 0.667 and reached statistical significance at p < 0.001, confirming that each observed variable exhibited high representativeness and explanatory power for its respective latent construct. [41,46] The squared multiple correlations (SMC) also fell within an acceptable range, indicating good indicator stability within the model.

**Table 3:** Convergent validity test table.

Dimensions	Question	Significance Estimate				Item Reliability		Component Reliability	Convergent Validity
		UnStd.	S.E.	z-value	P	Std.	SMC	CR	AVE
Task TechnologyFit	Q5	1				0.974	0.949	0.947	0.781
	Q6	0.699	0.042	16.58	***	0.777	0.604		
	Q7	0.87	0.035	24.924	***	0.894	0.799		
	Q8	0.857	0.032	26.956	***	0.912	0.832		
	Q9	0.873	0.042	20.873	***	0.849	0.721		
Role Adaptation	Q10	1				0.934	0.872	0.918	0.695
	Q11	0.673	0.057	11.735	***	0.667	0.445		
	Q12	0.947	0.044	21.711	***	0.899	0.808		
	Q13	0.879	0.04	22.005	***	0.903	0.815		
	Q14	0.785	0.057	13.773	***	0.733	0.537		
Self Efficacy	Q15	1				0.919	0.845	0.954	0.807
	Q16	0.849	0.044	19.202	***	0.865	0.748		
	Q17	0.918	0.043	21.269	***	0.899	0.808		
	Q18	0.919	0.043	21.559	***	0.903	0.815		
	Q19	0.922	0.042	21.725	***	0.906	0.821		
Institutional Support	Q20	1				0.903	0.815	0.951	0.795
	Q21	0.83	0.044	19.019	***	0.877	0.769		
	Q22	0.919	0.047	19.717	***	0.89	0.792		
	Q23	0.901	0.045	20.114	***	0.897	0.805		
	Q24	0.911	0.046	19.804	***	0.892	0.796		
Exploration Behavior	Q25	1				0.912	0.832	0.941	0.762
	Q26	0.789	0.051	15.363	***	0.789	0.623		
	Q27	0.932	0.047	19.961	***	0.889	0.790		
	Q28	0.869	0.045	19.217	***	0.876	0.767		
	Q29	0.924	0.046	20.251	***	0.894	0.799		
Exploitation Behavior	Q30	1				0.905	0.819	0.948	0.786
	Q31	0.799	0.048	16.823	***	0.828	0.686		
	Q32	0.96	0.045	21.383	***	0.916	0.839		
	Q33	0.895	0.043	20.933	***	0.909	0.826		
	Q34	0.91	0.048	18.948	***	0.873	0.762		
Learning Effect	Q35	1				0.906	0.821	0.958	0.818
	Q36	0.911	0.045	20.217	***	0.894	0.799		
	Q37	0.935	0.045	20.601	***	0.9	0.810		
	Q38	0.902	0.042	21.623	***	0.917	0.841		
	Q39	0.961	0.046	20.983	***	0.906	0.821		

***p < 0.001.

To assess convergent validity, the average variance extracted (AVE) was calculated for each construct. All AVE values exceeded 0.69, with the highest being for learning performance (AVE = 0.818) and the lowest for role adaptation (AVE = 0.695). These results indicate that each set of items shared a high level of variance, effectively capturing the underlying latent construct, and met the standard criteria for convergent validity. [45] In summary, the measurement instrument demonstrated excellent reliability and convergent validity, providing a robust foundation for subsequent structural model fitting and path analysis.

To ensure the construct validity of the measurement instrument, this study evaluated both convergent validity and discriminant validity. All validity indicators were assessed based on the measurement model results obtained through Structural Equation Modeling (SEM), using the criteria established by Fornell and Larcker. [45] Convergent validity was assessed using the Average Variance Extracted (AVE) for each latent variable. A higher AVE indicates that the construct captures more variance from its observed indicators. According to Fornell and Larcker, [45] an AVE value above 0.50 indicates acceptable convergent validity. In this study, all AVE values exceeded the 0.50 threshold, with the lowest being for Role Adaptation (AVE = 0.695). Other constructs, such as Learning Performance(AVE = 0.818), Institutional Support (AVE = 0.795), and Self-Efficacy (AVE = 0.807), all exceeded 0.76, indicating strong convergent validity and high internal consistency across measurement items.

Discriminant validity was examined using the Fornell-Larcker criterion, which requires that the square root of the AVE for each latent construct be greater than its correlations with any other construct. As shown in Table 4, the diagonal values represent the square roots of the AVEs and are all higher than the inter-construct correlation coefficients in the corresponding rows and columns. For example, the highest correlation between Learning Performance and another construct was 0.487 (with Self-Efficacy), while its AVE square root was 0.904, significantly higher than the correlation value. Similarly, constructs such as Task–Technology Fit (√AVE = 0.884), Institutional Support (0.892), and Exploration Behavior (0.873) also met the criterion.Therefore, all constructs successfully passed the Fornell-Larcker test, indicating strong discriminant validity. The findings confirm that each latent variable is sufficiently distinct and that the measurement scale is capable of accurately capturing different psychological or behavioral characteristics.

**Table 4:** Discriminant validity table.

	Convergent Validity	Discriminant validity						
	AVE	Learning Effect	Institutional Support	Self Efficacy	Role Adaptation	Task TechnologyFit	Exploitation Behavior	Exploration Behavior
Learning Effect	0.818	**0.904**						
Institutional Support	0.795	0.27	**0.892**					
Self Efficacy	0.807	0.487	0.189	**0.898**				
Role Adaptation	0.695	0.334	0.251	0.484	**0.834**			
Task TechnologyFit	0.781	0.281	0.231	0.4	0.386	**0.884**		
Exploitation Behavior	0.786	0.39	0.329	0.483	0.494	0.418	**0.887**	
Exploration Behavior	0.762	0.263	0.294	0.395	0.438	0.392	0.146	**0.873**

**Note**: The bold numbers on the diagonal are the square root of AVE, and the lower triangle is the Pearson correlation coefficient of the variables.

### 4.5. Model fit evaluation

The overall model fit of the proposed structural equation model was assessed, and the results indicated a good fit between the theoretical framework and the empirical data (Table 5). Specifically, the CMIN/DF (χ²/df) value was 2.185, below the recommended threshold of 3. The CFI, IFI, and TLI all exceeded 0.90, while the RMSEA and SRMR were 0.076 and 0.068, respectively—both falling below the 0.08 cutoff. These results suggest that the Exploration–Exploitation–Adaptation Model proposed in this study demonstrated acceptable fit indices and possesses solid structural stability and explanatory power.

**Table 5:** Model fit indicators.

Index	Model Value Indicator	Standard	Conclusion	Standard Source
CMIN	1188.471	The smaller the better		
DF	544	The smaller the better		
CMIN/DF	2.185	<3	Good fit	[47]
CFI	0.918	>0.9	Good fit	[49]
IFI	0.918	>0.9	Good fit	[51]
TLI(NNFI)	0.91	>0.9	Good fit	[46]
RMSEA	0.076	<0.08	Good fit	[46]
SRMR	0.068	<0.08	Good fit	[48]

The model in this study includes 7 latent variables and 10 hypothesized paths, representing a moderately complex SEM model. [46] The selected cutoff values were not simply applied from general standards but were chosen to suit the complexity of the model. In models with multiple constructs and paths, correlations among latent variables and the accumulation of residuals in observed variables increase the difficulty of achieving good fit. Therefore, thresholds of CMIN/DF < 3 [47] and RMSEA and SRMR < 0.08 [48] were adopted, balancing the need to avoid overly stringent fit requirements while effectively validating the structural rationality of the model. Cutoffs of CFI, IFI, and TLI > 0.9 [49] were applied to accurately assess the correspondence between latent and observed variables in a complex model, ensuring that construct measurement and path specifications were not biased due to model complexity.

This study focuses on reporting six widely recognized model fit indices: CMIN/DF, CFI, IFI, TLI, RMSEA, and SRMR, as they comprehensively capture model fitness from the perspectives of residual fit, comparative fit, and approximation error. These indices are considered the core metrics in contemporary SEM research. [46,48] In contrast, GFI and AGFI, although frequently used in earlier SEM studies, have been gradually de-emphasized in recent empirical research due to their high sensitivity to sample size and the lack of widely accepted theoretical cutoff criteria. [50] Therefore, to ensure the scientific rigor and comparability of model evaluation, this study does not report GFI and AGFI as primary fit indices.

### 4.6. Structural model validation

**Fig 3:**
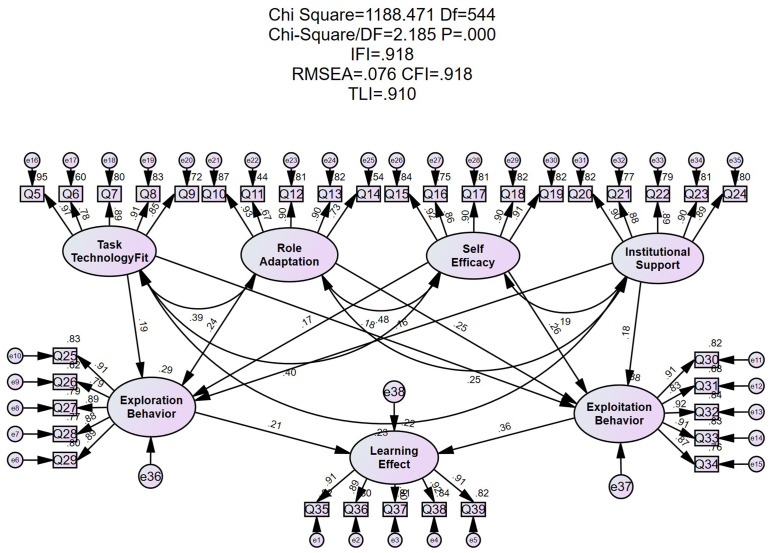
Structural equation model diagram.

This study validated the structural model using AMOS 26.0, focusing primarily on effect sizes (standardized path coefficients) and explained variance (R²) to reflect the substantive importance of variable relationships. Statistical significance tests were conducted only as a supplementary measure, avoiding equating statistical significance with substantive importance. The results are illustrated in Fig 3. According to the model estimation outcomes, all ten proposed hypotheses (H1 to H10) reached statistical significance and were supported. Detailed standardized path coefficients are presented in Table 6. Specifically, Task–Technology Fit, Role Adaptation, Self-Efficacy, and Institutional Support all exhibited significant positive effects on both Exploration Behavior and Exploitation Behavior. In turn, both types of behavior showed direct positive impacts on Learning Performance. These findings indicate that the proposed theoretical model is structurally sound, and the relationships among the latent variables are statistically meaningful.

**Table 6:** Model path analysis results.

	Unstd.	S.E.	C.R.	P	Std.	R^2^
Task Technology Fit--- > Exploration Behavior	0.16	0.059	2.718	0.007	0.194	0.293
Role Adaptation--- > Exploration Behavior	0.216	0.07	3.101	0.002	0.238	
Self Efficacy--- > Exploration Behavior	0.152	0.066	2.282	0.022	0.173	
Institutional Support--- > Exploration Behavior	0.143	0.06	2.361	0.018	0.156	
Task Technology Fit--- > Exploitation Behavior	0.152	0.058	2.616	0.009	0.175	0.382
Role Adaptation--- > Exploitation Behavior	0.244	0.069	3.539	***	0.255	
Self Efficacy--- > Exploitation Behavior	0.24	0.066	3.628	***	0.259	
Institutional Support--- > Exploitation Behavior	0.169	0.06	2.827	0.005	0.176	
Exploration Behavior--- > Learning Effect	0.227	0.076	2.968	0.003	0.208	0.223
Exploitation Behavior--- > Learning Effect	0.372	0.074	5.06	***	0.36	

In terms of path strength (see Table 6), Role Adaptation (Std. = 0.238) and Task–Technology Fit (Std. = 0.194) had relatively stronger effects on Exploration Behavior, while Role Adaptation (Std. = 0.255) and Self-Efficacy (Std. = 0.259) showed stronger effects on Exploitation Behavior. By contrast, the effects of Task–Technology Fit and Institutional Support on both types of behavior were comparatively weaker. The standardized path coefficients from Exploration Behavior and Exploitation Behavior to Learning Performance were 0.208 and 0.360, respectively, indicating that Exploitation Behavior had a more substantial impact on learning outcomes.

In addition, the R² values indicate good explanatory power for the endogenous variables: Exploration Behavior and Exploitation Behavior had R² values of 0.293 and 0.382, respectively, while the R² for Learning Performance was 0.223. These results demonstrate that the model has strong explanatory capability for both behavioral and outcome variables. Thus, the proposed Exploration–Exploitation–Adaptation model exhibits structural stability and clearly defined path relationships, thus providing correlational insights into potential pathways of GAI-assisted learning in higher education contexts. The core framework of this study aims to validate the mediating transmission effect of the dual behaviors. No other indirect effect hypotheses were preset; therefore, indirect effects were not specifically tested using methods such as bootstrapping. Instead, the overall plausibility of this chained pathway was assessed through the direct effect sizes of each path and the R² values.

## 5. Discussion and conclusion

### 5.1. Discussion on the collaborative mechanisms of gai in higher education

This study, grounded in the “exploration–exploitation–adaptation” triadic behavioral framework and a human–AI collaboration perspective, elucidates the behavioral response logic of higher education learners in GAI-assisted learning contexts. In contrast to the empirical presentation in Section 4, this subsection focuses on a deeper theoretical interpretation of the core mechanisms, with particular emphasis on explaining why role adaptation consistently occupies a dominant position across both behavioral pathways.

The centrality of role adaptation fundamentally arises from the need to reconstruct learner agency in human–AI collaborative learning environments. In traditional technology-assisted learning, learners typically occupy a passive role as “tool users.” By contrast, GAI, as a “cognitive collaborator”, [7] transforms the instructional structure from a binary “teacher–student” relationship into a triadic “teacher–agent–student” configuration. [11] This structural shift requires learners to transition from “knowledge recipients” to “co-constructors.” Only when learners clearly define their responsibilities and boundaries within this collaborative relationship can they move beyond superficial tool use and engage in deeper interaction. Role adaptation represents a process of cognitive reconstruction of one’s self-positioning in a new environment. Such reconstruction directly shapes the intensity and direction of behavioral motivation. When learners successfully adapt to their collaborative role in GAI-assisted scenarios, they develop a cognitive frame of “learning tasks are completed jointly by me and the AI,” which reduces psychological resistance to exploring new functions and enhances proactive integration of technology into task execution.

Within the exploration pathway, the dominance of role adaptation is manifested in its capacity to break entrenched learning patterns. Exploration behavior centers on transcending unknown functionalities and knowledge boundaries, which requires learners to abandon the traditional perception of technology as merely an auxiliary tool. Role adaptation enables learners to perceive GAI as a “partner in exploration” rather than a simple functional carrier. This cognitive shift motivates them to actively experiment with diverse prompts and to explore cross-domain applications—hallmark features of exploration behavior. Compared with task–technology fit and self-efficacy, role adaptation addresses the more fundamental motivational question of “why explore,” thereby becoming the most critical driver of exploratory behavior.

Within the exploitation pathway, the dominance of role adaptation derives from its function in enabling effective collaboration. Exploitation behavior emphasizes the deep integration of GAI with concrete learning tasks, requiring learners to use technology efficiently while maintaining cognitive primacy. Role adaptation clarifies that “AI is an assisting means, while the learner remains the core agent of task completion.” This sense of responsibility prevents overreliance on AI and the emergence of “cognitive inertia,” instead promoting precise alignment between technology and task demands. In this process, role adaptation and self-efficacy operate in a complementary manner: self-efficacy ensures the capability to “use AI well,” whereas role adaptation delineates the behavioral boundaries of “how to use AI appropriately.” Together, they facilitate the effective enactment of exploitation behavior.

The dominance of role adaptation does not negate the contributions of other variables. Task–technology fit provides the “instrumental feasibility” of collaboration, and institutional support lowers the “environmental threshold.” However, these external conditions must ultimately be transformed through role adaptation as an “internal cognitive bridge” in order to generate sustained exploration and exploitation behaviors. This mechanism resonates with the core characteristics of human–AI collaborative education: the value of GAI does not reside solely in its technical functionalities but depends on whether learners can achieve cognitive complementarity with technology through role reconstruction [6]. This, in turn, constitutes the theoretical essence of “adaptation” as the central connective dimension within the triadic framework.

A recent series of studies on the context of higher education in Palestine [52–57] have explored key dimensions such as AI dependency, technostress, ethical tensions, and subjectivity construction from qualitative, phenomenological, psychoanalytic, and extended technology acceptance perspectives. These studies provide cross-cultural contextual support and a psychological-level complement to the present quantitative research.

### 5.2. Discussion on the effectiveness of GAI in higher education

Structural equation modeling based on cross-sectional data indicates that both Exploration Behavior and Exploitation Behavior are significantly and positively associated with learning outcomes, though with differing strengths: the standardized association coefficient for Exploitation Behavior (0.360) is higher than that for Exploration Behavior (0.208). It is important to note that “learning Effect” in this study are measured through self-reports and therefore reflect learners’ perceived learning performance rather than objective learning achievements. The structural model further shows that Exploration and Exploitation behaviors together explain only 22.3% of the variance in perceived learning performance, leaving 77.7% of the variance unexplained by the current model. This suggests that additional factors—such as learners’ prior knowledge, instructional guidance provided by teachers, and heterogeneity in the functional design of GAI tools—may constitute important alternative mechanisms. The pathways through which these factors operate warrant further investigation in future research.

In the path analysis of GAI’s impact on learning outcomes, the results show that both exploration and exploitation behaviors exert significant positive effects. However, there is a notable difference in the strength of these effects. The standardized path coefficient for exploitation behavior is 0.360, considerably higher than that of exploration behavior (0.208). This indicates that, within GAI-assisted learning environments, students’ effective integration of GAI into specific learning tasks is more closely associated with learning effects—consistent with the cross-sectional, self-reported data characteristics and observed effect sizes of this study. The findings also indicate that exploitation behavior is more closely associated with learning effects. In other words, remaining at the level of mere experimentation or initial contact yields only limited benefits. It is the deep integration of GAI tools into the learning process—the “effective utilization”—that serves as the critical pathway to enhancing learning performance.

Although the influence of exploration behavior is relatively weaker, its positive role should not be overlooked. Through preliminary use of GAI for information retrieval, knowledge expansion, and idea generation, learners can activate motivation and cognitive engagement, laying the psychological and skill-based foundation for subsequent advanced learning behaviors. However, if exploration does not evolve into effective exploitation, its value may remain confined to surface-level operations. Therefore, exploration behavior should be understood as a precursor and transitional stagetoward exploitation, rather than an end in itself. Moreover, the structural model’s R² values indicate that exploration behavior and exploitation behavior explain 29.3% and 38.2% of their variance, respectively, while the R² for learning outcomes is 22.3%. These results demonstrate that the proposed model possesses strong explanatory and predictive power. GAI does not influence learning outcomes as a standalone tool; rather, it exerts its effect by activating behavioral pathways, enhancing learning engagement, and improving task efficiency—In statistical terms, it exhibits a considerable impact effect. Given the modest R² value (22.3%) for learning effects and the study’s reliance on self-reported cross-sectional data, the potential practical relevance of these findings should be interpreted cautiously.

In conclusion, The learning-related value of GAI in higher education appears to go beyond its convenience as a tool. Its observed utility is associated with shifts in learning behaviors—specifically, self-reported increases in students’ proactive and efficient engagement with cognitive and skill-based tasks—consistent with the correlational patterns identified in the study’s self-reported data.This highlights the critical importance of promoting the effective utilization of GAI among students, rather than mere passive exposure.

### 5.3. Conclusion and future directions

The integration of Generative Artificial Intelligence (GAI) into higher education is not only reshaping how knowledge is acquired and tasks are accomplished, but is also profoundly influencing learners’ behavioral trajectories, cognitive mechanisms, and pathways to learning outcomes. Grounded in the “Exploration–Exploitation–Adaptation” triadic framework, this study systematically validated the functional mechanisms of GAI in learning processes. It explicitly revealed the synergistic effects of individual psychological factors (e.g., role adaptation and self-efficacy) and external technological environment variables (e.g., task–technology fit and institutional support) in driving human–AI collaborative learning behaviors.From a mechanistic perspective, the effective embedding of GAI hinges on whether learners can undergo identity transformation, proactively adapt to new roles, and engage in both exploratory and exploitative behaviors at cognitive and operational levels. From an outcome perspective, the achievement of learning outcomes is more strongly associated with students’ transition from “trial-based use” to “task-integrated application” of GAI tools—consistent with the correlational patterns observed in the study’s data.

The specific contributions of this study are threefold. First, it clarifies a “dual-driven mechanism” of GAI-supported collaborative learning, in which Role Adaptation and Self-Efficacy function as core internal psychological drivers, while Task–Technology Fit and Institutional Support serve as key external environmental enablers; together, these forces promote the emergence of both Exploration and Exploitation behaviors. Second, it is found that both types of behaviors are significantly and positively associated with learners’ perceived learning performance, with exploitation behavior showing a stronger correlational link — a finding that reflects the close connection between task-integrated use of GAI and learners’ perceived learning experiences. Third, the study constructs and empirically validates an “Adaptation–Behavior–Outcome” pathway model, providing an operational framework for elucidating the behavioral logic of human–AI collaborative learning.

Nevertheless, this study has several limitations. First, self-report bias. All core variables were measured through learners’ subjective reports, which may be influenced by social desirability and individual cognitive preferences, and thus may not fully reflect objective learning outcomes. Although procedural remedies were implemented to mitigate common method bias, reliance on a single data source may still affect the objectivity of the observed relationships. Second, the single-institution context. All participants were drawn from universities in China, where GAI resource allocation, instructional practices, and institutional support exhibit contextual particularities. The absence of comparisons across countries, institution types, and levels of educational resources limits the generalizability of the findings and constrains their direct transferability to other educational settings. Third, the cross-sectional design precludes capturing the dynamic evolution of variables over time and prevents examination of long-term relationships between learning behaviors and outcomes. Moreover, the heterogeneous effects of different types of GAI tools were not sufficiently explored.

Future research may extend this work in three directions. First, adopting mixed-method approaches that integrate self-report data with objective indicators can reduce subjective bias and more accurately capture the actual educational value of GAI. Second, expanding the sample to include learners from diverse regions and institution types, and incorporating teacher-related variables, would enable the construction of a “teacher–student–GAI” triadic interaction model to examine the moderating role of instructional guidance. Third, longitudinal designs should be employed to observe the dynamic evolution of the “Exploration–Exploitation–Adaptation” process over time and to differentiate the contextual applicability of various GAI functions, thereby clarifying the boundary conditions under which these mechanisms operate and providing more targeted guidance for the optimization of intelligent educational systems.

## Supporting information

S1 File Raw dataset used for all analyses in this study. The file is provided in Excel format (.xlsx) and includes all variables and observations reported in the manuscript.
(XLSX)
